# Solvent effects on the kinetics of 4-nitrophenol reduction by NaBH_4_ in the presence of Ag and Au nanoparticles†

**DOI:** 10.1039/d2re00044j

**Published:** 2022-04-29

**Authors:** Vladimir Lomonosov, Jérémie Asselin, Emilie Ringe

**Affiliations:** Department of Materials Science and Metallurgy, University of Cambridge 27 Charles Babbage Road Cambridge CB3 0FS UK er407@cam.ac.uk +44 (0)1223 334567 +44 (0)1223 334330; Department of Earth Sciences, University of Cambridge Downing Street Cambridge CB2 3EQ UK

## Abstract

The reduction of 4-nitrophenol (4-NiP) to 4-aminophenol (4-AP) with an excess of sodium borohydride is commonly used as a model reaction to assess the catalytic activity of metallic nanoparticles. This reaction is considered both a potentially important step in industrial water treatment and an attractive, commercially relevant synthetic pathway. Surprisingly, an important factor, the role of the reaction medium on the reduction performance, has so far been overlooked. Here, we report a pronounced effect of the solvent on the reaction kinetics in the presence of silver and gold nanoparticles. We demonstrate that the addition of methanol, ethanol, or isopropanol to the reaction mixture leads to a dramatic decrease in the reaction rate. For typical concentrations of reactants, the reduction is completely suppressed in the presence of 50 vol% alcohols. 4-NiP reduction rate in aqueous alcohol mixtures can, however, be improved noticeably by increasing the borohydride concentration or the reaction temperature. The analysis of various factors responsible for solvent effects reveals that the decrease in the reduction rate in the presence of alcohols is related, amongst others, to a substantially higher oxygen solubility in alcohols compared to water. The results of this work show that the effects of solvent properties on reaction kinetics must be considered for unambiguous comparison and optimization of the catalytic performance of metallic nanoparticles in the liquid phase 4-NiP reduction.

## Abstract 2

The presence of methanol, ethanol, or isopropanol in the reaction mixture substantially affects the kinetics of 4-nitrophenol reduction in aqueous medium.

## Graphical abstract

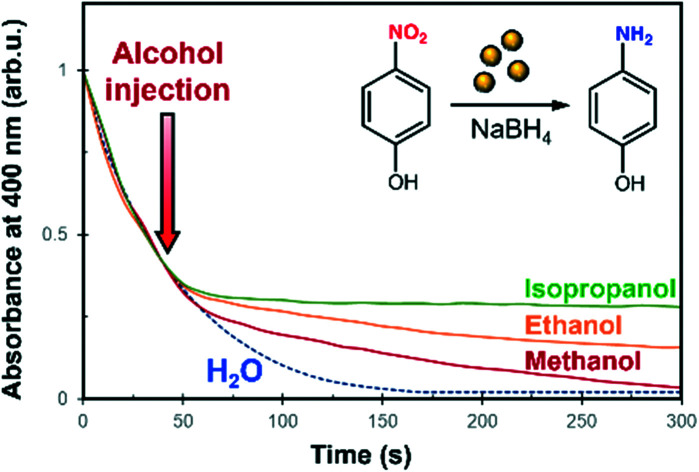

## Introduction

Liquid phase, heterogeneously catalysed reactions are the basis of many industrially important processes including fine chemicals production, pharmaceutical manufacturing, and industrial waste treatment. Most of such processes are performed in a large amount of solvent to dissolve solid reactants, decrease viscosity, or control reaction rate and temperature. It is generally accepted that solvents play a critical role in heterogeneous catalysis.^1–4^ The importance of the solvent is mainly determined by two factors: (1) being the main component of the reaction medium, the solvent and its physical properties (viscosity, polarity, ability to dissolve gaseous compounds, *etc.*) impact the reaction kinetics by affecting mass and heat transfer, and (2) the solvent can also chemically or physically interact with the reaction components (catalyst, substrates, or products). Indeed, solvents in liquid phase heterogeneous catalysis can rarely be considered absolutely inert, hence their parameters inevitably affect catalytic performance. It is therefore not surprising that the pronounced effects of solvent have been reported for several types of heterogeneous catalytic processes including hydrogenation,^4–6^ oxidation,^7,8^ and cross-coupling^9–11^ reactions. A comprehensive review of the solvent effects in catalysis can be found in a recent paper.^2^ Despite its obvious importance, the role of the reaction medium in heterogeneous catalytic processes is often overlooked and remains an area ripe for significant advancements.

Metal nanoparticles (NPs) are central to heterogeneous catalysis research owing to the unique properties of nanomaterials.^12–14^ Their substantially higher surface-to-volume ratio than that of bulk materials provides a larger number of active sites per volume, and potentially different sites such as edges and corners, resulting in enhanced catalytic performance. In the last decade, liquid phase reduction of 4-nitrophenol (4-NiP) to 4-aminophenol (4-AP) with an excess of NaBH_4_ has become a “gold standard” for evaluating the catalytic performance of metallic NPs. The reaction proceeds efficiently in an aqueous medium and is characterised by an absence of side products, the simplicity of the experimental procedure, and a minimal instrumentation requirement as it can be tracked by UV-vis spectroscopy.^15,16^

While aqueous 4-NiP reduction is widely used to evaluate and compare the performance of different NPs,^17–20^ to the best of our knowledge, solvent effects on the reaction kinetics have not been investigated. This is a significant knowledge gap for at least three reasons. First, although the 4-NiP reduction mechanism has been extensively studied, some of its aspects are still not fully understood,^16,21,22^ and studying solvent effects can shed more light onto the reaction mechanism. Second, 4-NiP reduction with NaBH_4_ is considered a potentially important step in industrial water treatment: 4-NiP is an environmentally toxic by-product of many processes including agrochemistry, dye manufacturing, and pharmaceutical production. The discharged industrial wastewater may include different organic solvents and wastewater treatment processes might involve the efficiency and speed-enhancing addition of small carbon sources such as methanol, ethanol, or acetic acid.^23,24^ Third, 4-AP is a key intermediate in the synthesis of several analgesics and antipyretic drugs such as paracetamol, acetanilide, and phenacetin,^25,26^ making 4-NiP reduction by NaBH_4_ a potentially attractive and commercially relevant synthetic pathway. Together, these make systematic data on the correlation between the properties of the reaction medium and catalytic performance of interest, with potential impact on reaction optimization *via* solvent design.

Here we present the results of a comprehensive experimental study of the effect of the reaction medium on the kinetics of 4-NiP reduction with an excess of NaBH_4_ in the presence of either of the common catalysts Ag or Au NPs.^18,27,28^ We use an array of solvents including water, methanol (MeOH), ethanol (EtOH), isopropanol (IPA), and their mixtures to assess how the solvent composition modifies the reaction kinetics across a wide range of experimental conditions such as different 4-NiP and NaBH_4_ concentration, as well as various temperatures. The pronounced effect of the reaction medium on the reduction rate is reported and correlated with solvent properties.

## Experimental

### Nanoparticle synthesis and characterization

Citrate-capped Au NPs were synthesised using a modified Turkevich methodology described by Schulz *et al.*^29^ Briefly, 80 mL of a 2.75 mM citrate buffer was prepared by dissolving 48.5 mg of sodium citrate tribasic dihydrate, 10.6 mg of citric acid, and 0.6 mg of ethylenediaminetetraacetic acid disodium salt dihydrate in deionised (DI) water, and another 20 mL of DI water was used to dissolve 6.4 mg of hydrogen tetrachloroaurate trihydrate. The two solutions were brought to a boil under intense stirring for 10 minutes, before adding the HAuCl_4_ solution to the buffered mixture and leaving to react for 20 minutes. After allowing the mixture to cool down to room temperature, the colloidal suspension was diluted to 100 mL with DI water and stored at 4 °C before the catalytic experiments.

Citrate-capped Ag NPs were prepared with a size-controlled seeded-growth protocol previously optimised by Asselin *et al.*^30^ The seed solution was prepared by heating 95 mL of a 7.2 mM sodium citrate tribasic solution at 70 °C, and then adding successively 1.7 mL of a silver nitrate solution (59 mM) and 0.2 mL of a fresh sodium borohydride mixture (264 mM). This reaction mixture was left to react for 30 minutes before diluting it to 100 mL with DI water. In a round-bottom flask equipped with a water cooler, 10 mL of this Ag NPs suspension was added to 75 mL DI water and brought to reflux, before the successive additions of 2.0 mL of sodium citrate (34 mM) and 1.7 mL of silver nitrate solution (59 mM). This mixture was left to stir for 1 hour before cooling down to room temperature. The 20 nm Ag NPs were then diluted to 100 mL, and 10 mL of the resulted solution was further diluted with 80 mL DI water and heated to 88 °C under stirring. After successively adding 2.0 mL of sodium citrate (34 mM) and 1.7 mL of silver nitrate (59 mM), this final growth step was left to proceed for 2 hours. This colloidal suspension was diluted to 100 mL using DI water and stored at 4 °C before the catalytic experiments.

**Fig. 1:**
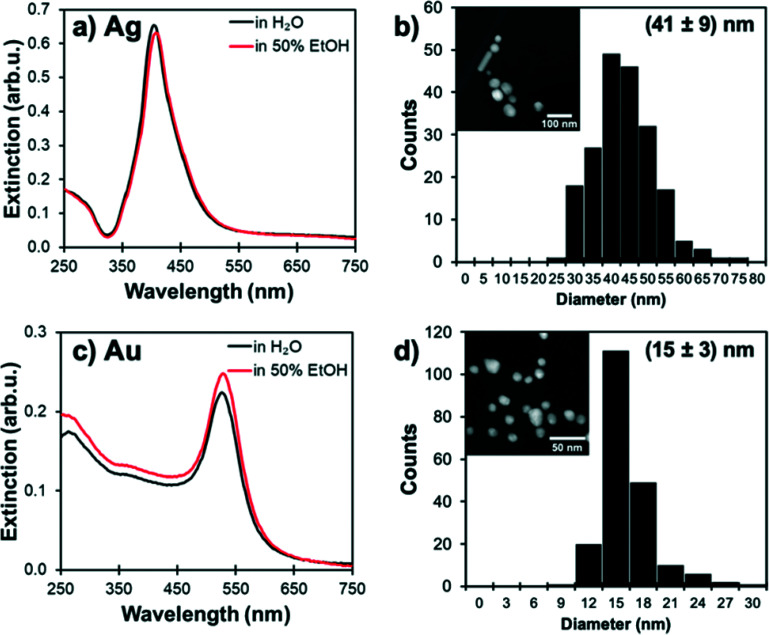
Size distribution and plasmonic properties of Ag and Au NPs. Extiction spectrum of (a) Ag and (c) Au NPs dispersed in water and 50 vol% water–ethanol mixture and STEM images (insets) and histograms of the size distribution of the Ag (b) and Au (d) NPs.

The plasmonic extinction of Au NPs and Ag NPs suspensions was characterised by UV-vis spectroscopy, and their size distribution was measured by scanning transmission electron microscopy (STEM) (Fig. 1). Au NPs were found to have an extinction maximum at 530 nm and an average size of 15 nm, while the Ag NPs had an extinction maximum at 410 nm and an average size of 42 nm. The stability of Ag and Au NPs in solvents was confirmed by UV-vis spectroscopy; no changes in the optical spectra were observed over 30 minutes for 0.1 mL of freshly prepared NPs mixed with 1 mL of water and 50 vol% aqueous alcohol mixture.

### 4-NiP reduction experimental procedure

The catalytic 4-NiP reduction was carried out in a fused quartz cuvette with a 1 cm path length (ThorLabs) under continuous stirring. The reaction progress was monitored by UV-vis spectroscopy (ThermoFisher Evolution™ 220 with a temperature-controlled cuvette holder), detecting the decrease in the absorbance peak at 400 nm attributed to the 4-nitrophenolate anion.^15,16^ Typical time-dependent absorption spectra demonstrating the evolution of the 4-aminophenol peak and the decay of the 4-nitrophenolate anion peak during the 4-NiP reduction is shown in Fig. S1.† Given that the reaction kinetics is a pseudo-first-order with respect to 4-NiP concentration,^16,31,32^ an apparent reaction rate constant, *k*_app_, was calculated from the linear slope of the −ln(*A*/*A*_0_) *versus* time plot, where *A*/*A*_0_ is the normalized 4-NiP absorbance. Note that the extinction by the Au and Ag NPs at 400 nm was negligible compared to 4-nitrophenolate anion due to the substantial concentration differences (Fig. S2†).

In a typical experiment, the 4-NiP and the freshly prepared NaBH_4_ solutions were added sequentially to the cuvette and the reaction was initiated by injecting a catalytic NP suspension into the solution.

### 4-NiP reduction experimental conditions

The qualitative effect of alcohols on the 4-NiP reduction rate over Au and Ag NPs was studied by the addition of 25 vol% of methanol, ethanol, or isopropanol to the reaction mixture. The corresponding alcohol was either in the reaction mixture initially or was injected in an aqueous medium 45 s after the reduction began. In both experiments, the total reaction volume was 1.5 mL and contained 0.1 mM of 4-NiP, and 15 mM of NaBH_4_. The amount of the catalyst in the final solution was selected based on preliminary experiments on 4-NiP reduction in water and was 7.0 × 10^−4^ and 4.0 × 10^−3^ mM of Au and Ag NPs, respectively (molarity reported with respect to Au and Ag atoms). At that concentration, full 4-NiP conversion was obtained several minutes after the catalyst was injected into the reaction medium.

More detailed studies of 4-NiP reduction in water–ethanol mixtures were performed over Ag NPs. The effects of various reaction parameters on the process kinetics were investigated as follows.

#### Alcohol content

The initial ethanol concentration was varied from 25 to 75 vol% at fixed initial concentrations of 4-NiP (0.1 mM) and NaBH_4_ (15 mM).

#### 4-NiP and NaBH_4_ concentrations

Two sets of experiments were carried out at three different ethanol content (25, 50, and 75 vol%); (1) 4-NiP concentration was varied in a range of 0.025 to 0.15 mM at constant concentrations of NaBH_4_ (15 mM); (2) the concentration of NaBH_4_ varied from 15 to 100 mM while the amount of 4-NiP was kept constant (0.1 mM).

#### Temperature

The 4-NiP reduction was performed using reaction mixtures containing 0.1 mM of 4-NiP and 15 mM of NaBH_4_ dissolved in water and 50 vol% water–ethanol mixture, and the reaction temperature was varied from 20 to 45 °C. Since the temperature dependence of reaction rate is not affected by the catalyst amount, the reduced concentration of Ag NPs was used to decrease the 4-NiP reduction rate in water and to maintain sufficient time resolution of the kinetic experiments at elevated temperatures. The concentration of Ag NPs in the reaction mixture was 1.5 × 10^−3^ and 4 × 10^−3^ mM for water and water–ethanol mixture, respectively.

#### pH of the reaction medium

First, a solution containing 0.1 mM of 4-NiP was prepared in an aqueous citrate buffer with pH varying from 4 to 6. Then, 0.7 mL of the resulting solution was mixed in a quartz cuvette with 0.7 mL of 15 mM NaBH_4_ dissolved in ethanol. The 4-NiP reduction was initiated by injection of 4.0 × 10^−3^ mM of Ag NPs. Note that pH of the final reaction mixture was affected by the addition of sodium borohydride and was between 8 and 9 depending on the pH of the citrate buffer.

#### Dissolved oxygen concentration

In this series of experiments, 15 mL of 4-NiP (0.1 mM) solution was added to a 25 mL round-bottom flask and purged with N_2_ flow (25 mL min^−1^) under vigorous stirring to remove dissolved oxygen. After 30 min of purging, 8.5 mg of NaBH_4_ was added, and after it dissolved, the 4-NiP reduction was initiated by the addition of 5.0 × 10^−4^ mM of Ag NPs. The reduction was carried out under continuous stirring and N_2_ purging to prevent O_2_ uptake from the atmosphere. Aliquots (0.5 mL) were taken every 2.5 min and absorbance at 400 nm was measured by UV-vis spectroscopy. Control experiments were performed without oxygen removal under the same conditions.

## Results

### General effect of alcohols on 4-NiP reduction rate over Ag and Au NPs

**Fig. 2:**
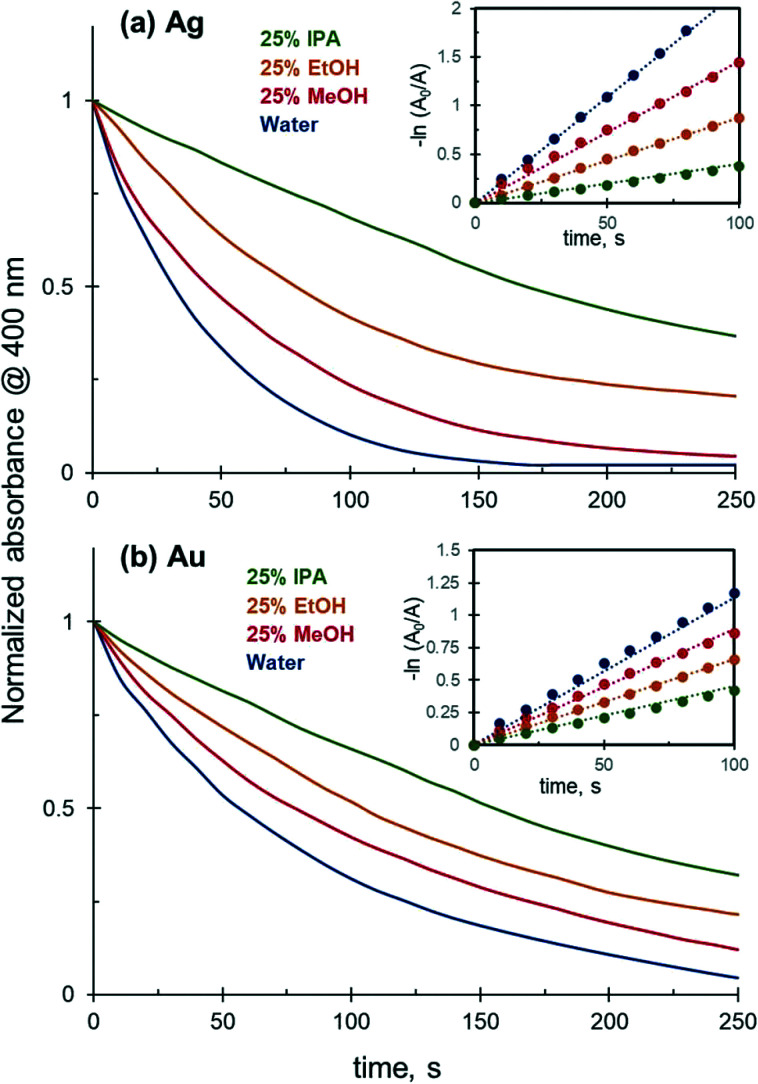
Effect of solvent composition on the 4-NiP reduction kinetics catalyzed by: (a) 4.0 × 10^−3^ mM of Ag NPs; (b) 7.0 × 10^−4^ mM of Au NPs.

Although the reaction between 4-NiP and NaBH_4_ is thermodynamically favourable, it does not occur in the absence of a catalyst due to the mutual repulsion of negatively charged 4NiP^−^ and BH_4_^−^ ions. Preliminary experiments in which a solution of 4-NiP and NaBH_4_ was left under stirring for 60 minutes indeed produced no detectable change in absorption at 400 nm, which corresponds to no reduction of the nitrophenolate anion. In contrast, the reduction of 4-NiP proceeds efficiently in an aqueous medium in the presence of Ag or Au NPs and can be tracked as a gradual decrease of the absorption at 400 nm, corresponding to the consumption of nitrophenolate anion with reaction time until the absorbance reaches zero (full 4-NiP conversion) after 150 and 250 s for the Ag and Au NPs, respectively (Fig. 2).

The reduction kinetics is strongly affected by the presence of alcohol in the reaction medium. The addition of 25 vol% of methanol, ethanol, or isopropanol to the initial mixture leads to a noticeable decrease in the 4-NiP reduction rate. For both Au and Ag NPs, the observed negative effect increases with the molecular weight of the corresponding alcohol (Fig. 2). 4-NiP reduction follows a pseudo-first-order kinetics both in water and in 25 vol% aqueous solutions of various alcohols (Fig. 2, insets). The values of the apparent rate constants extracted from the plots are reported in Table 1, showing that the inhibiting effect of alcohols is more pronounced for Ag than for Au NPs. For example, for Ag NPs the apparent rate constant in 25 vol% ethanol was nearly 2.5 times lower compared to that in water, while in the case of Au NPs the rate constant decreased only by a factor of 1.7.

**Table 1:** Apparent rate constants for the solvent-dependent 4-Nip reduction catalyzed by Ag NPs (4.0 × 10^−3^ mM) and Au NPs (7.0 × 10^−4^ mM). All solutions are aqueous

Catalyst	*k*_app_ × 10^3^, s^−1^			
	100% H_2_O	25% MeOH	25% EtOH	25% IPA
Ag NPs	21.8	14.6	8.8	4.0
Au NPs	11.3	8.9	6.6	4.5

**Fig. 3:**
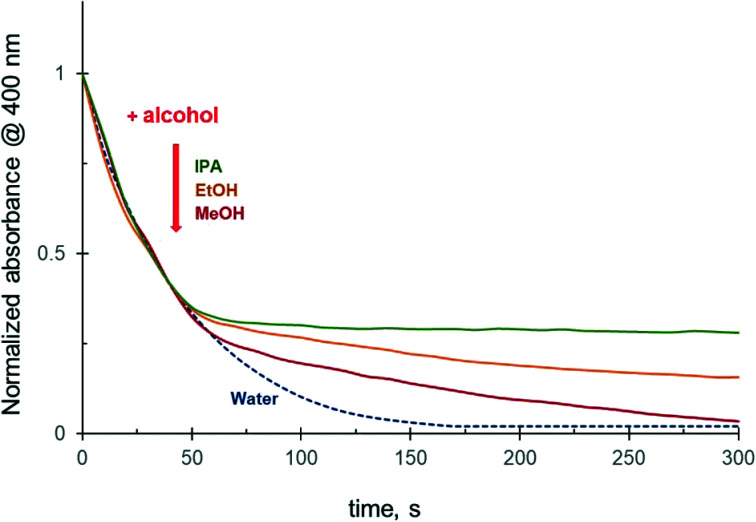
Effect of alcohol injection (at 45 s) into the reaction mixture on the 4-NiP reduction kinetics over Ag NPs. In both the main figure and the inset, water, methanol, ethanol and isopropanol are color-coded blue, red, yellow, and green, respectively.

To further investigate the effect of alcohols on the 4-NiP reduction kinetics, methanol, ethanol, or isopropanol was injected into the aqueous reaction mixture in the course of the reduction. The results show that the addition of alcohol during the 4-NiP reduction causes an essentially instant kinetic response characterized by a noticeable decrease of the slope of the kinetic curve (Fig. 3). Both methanol and ethanol injections lead to a substantial decrease in the observed 4-NiP reduction rate, while upon isopropanol addition the rate becomes close to zero (Fig. 3, inset).

### Effect of ethanol concentration on 4-NiP reduction kinetics over Ag NPs

**Fig. 4:**
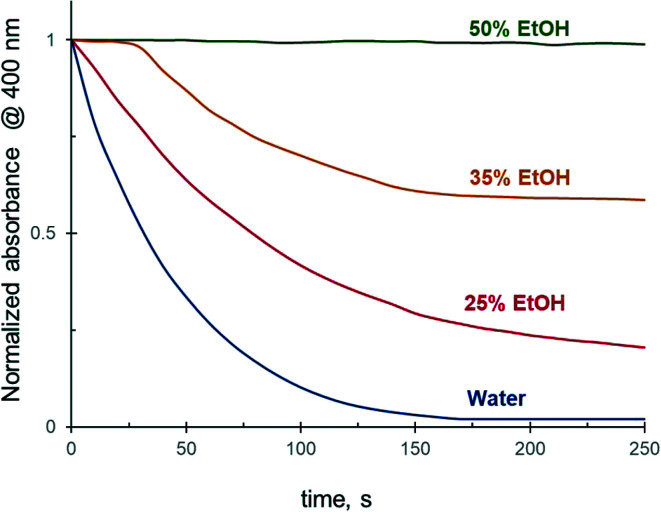
The effect of ethanol concentration in water on the reduction kinetics of 4-NiP in the presence of Ag NPs.

As shown above, the addition of 25 vol% of alcohols to the reaction medium substantially decreases the 4-NiP reduction rate. Experiments carried out in 25 to 75 vol% aqueous ethanol revealed that a further increase of the alcohol concentration in the initial mixture results in a more pronounced effect (Fig. 4). In the presence of 35 vol% of EtOH, for instance, the 4-NiP reduction kinetics is characterized by the appearance of an induction period, *i.e.* an initial time interval where no change in absorbance at 400 nm is detected (Fig. 4). After this interval, lasting approximately 30 s, the reaction proceeds slowly and 4-NiP conversion does not exceed 50% after 250 s. At higher ethanol content, 50 vol% (Fig. 4) and 75% (not shown), no detectable change in 4-NiP absorbance is observed, *i.e.* the reaction is completely inhibited.

### Effect of the concentration of reactants on 4-NiP reduction kinetics in water–ethanol mixtures

**Fig. 5:**
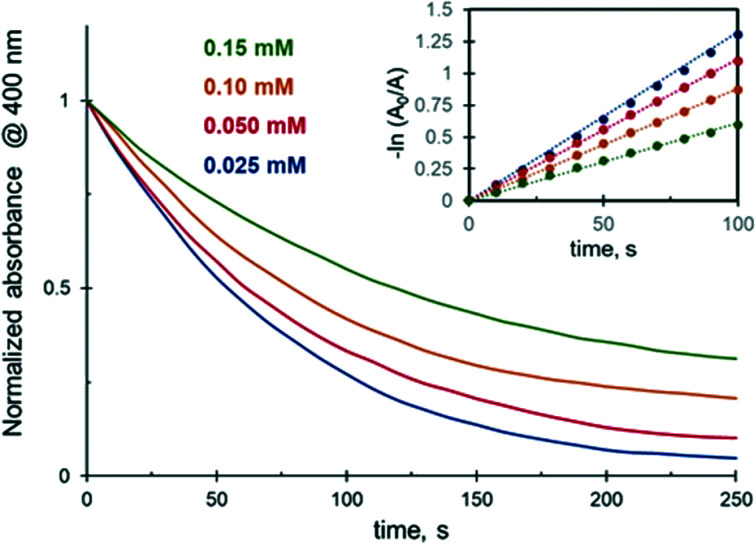
Effect of 4-NiP concentration on the 4-NiP reduction kinetics over Ag NPs in the presence of 25 vol% aqueous ethanol. In both the main figure and the inset, 0.025, 0.050, 0.10, and 0.15 mM 4-NiP are color-coded blue, red, yellow, and green, respectively.

Experimental results indicate that 4-NiP reduction in the presence of 25 vol% of EtOH slows down with the increase in initial 4-NiP concentration (Fig. 5). The apparent rate constant decreases 2.5 times with an increase in the concentration of 4-NiP from 0.025 mM to 0.15 mM (Table 2). A decrease of the reduction rate with increasing 4-NiP concentration is generally observed when the reaction is performed in aqueous medium.^32,33^ It is widely accepted that the reduction of 4-NiP proceeds *via* the Langmuir–Hinshelwood mechanism, which implies that the reaction occurs only between species adsorbed on the surface of the NPs.^32,34,35^ Since both reactants compete for the surface active sites, higher concentrations of 4-NiP, strongly adsorbing to the surface of metallic NPs, result in a lower number of active sites available for NaBH_4_ and thus slower reduction rates.

**Table 2:** Apparent rate constants (*k*_app_) for the 4-NiP reduction in the presence of 25 vol% aqueous ethanol over Ag NPs (7.0 × 10^−3^ mM) at different initial concentrations of 4-NiP in the reaction mixture

*k*_app_ × 10^3^, s^−1^			
0.025 mM	0.050 mM	0.10 mM	0.15 mM
13.3	11.1	8.8	5.2

**Fig. 6:**
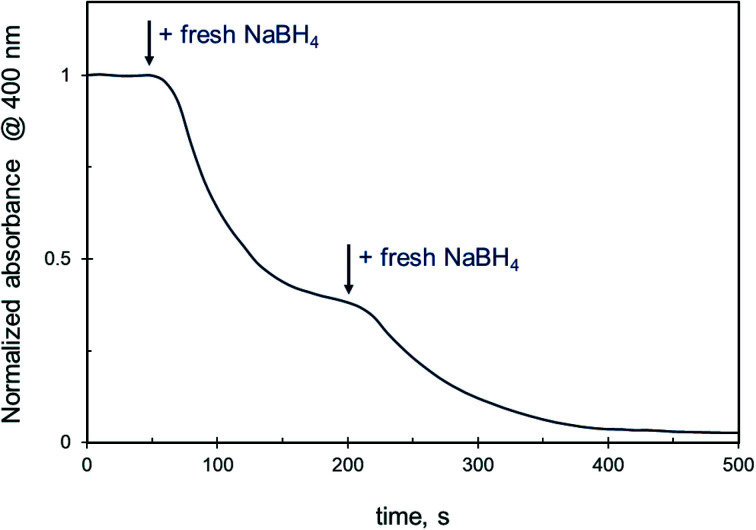
Effect of NaBH_4_ injection into the reaction mixture on the 4-NiP reduction kinetics over Ag NPs in the presence of 50% aqueous ethanol.

Under conditions when the reduction is completely suppressed by the presence of ethanol (*i.e.* ethanol content ≥50 vol%), the change in 4-NiP concentration does not affect the reaction. Moreover, neither an increase of initial catalyst up to 25 × 10^−3^ mM, nor a sequential addition of fresh catalyst during the reaction at such ethanol concentrations causes changes in the reaction rate. However, the stepwise introduction of additional freshly prepared NaBH_4_ solution to the reaction volume remarkably alters the reduction kinetics. The 4-NiP absorbance indeed decreases rapidly after NaBH_4_ is injected in the reaction medium containing 50 vol% aqueous ethanol (Fig. 6). Then, the slope of the kinetic curve decreases with time, and a subsequent injection of fresh NaBH_4_ leads to a further increase in the reaction rate.

**Fig. 7:**
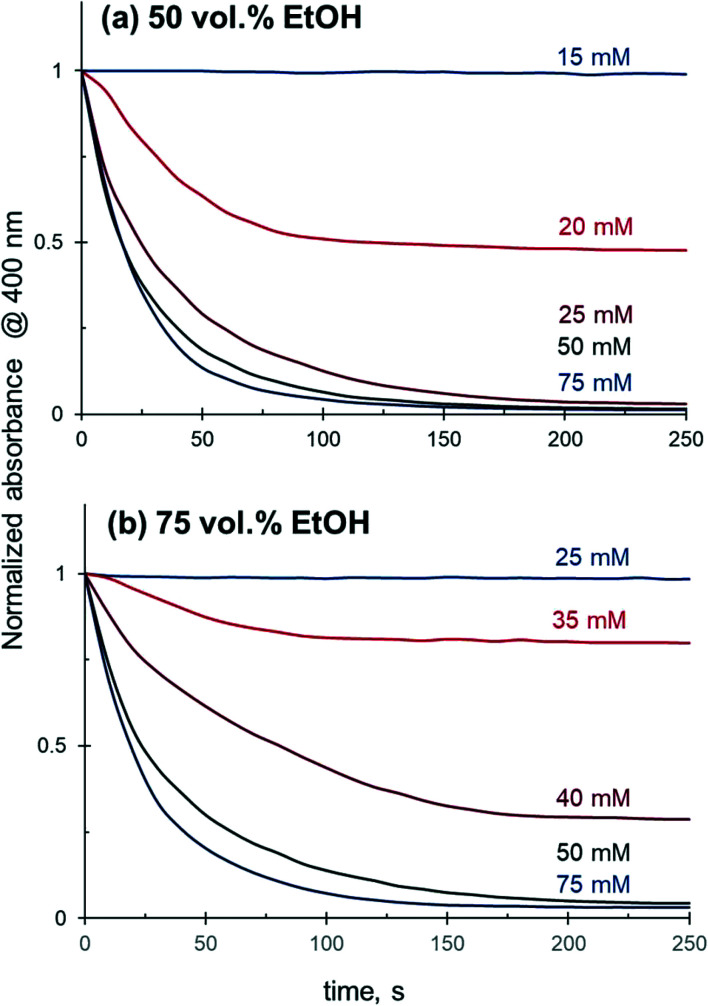
Effect of NaBH_4_ concentration on the 4-NiP reduction kinetics over Ag NPs in (a) 50 vol% and (b) 75 vol% aqueous ethanol.

Further investigation confirmed that 4-NiP conversion in aqueous alcohol is strongly affected by the initial concentration of NaBH_4_, where higher concentrations of NaBH_4_ lead to a more rapid 4-NiP reduction. Fig. 7 shows the time-dependence of 4-NiP consumption at different NaBH_4_ concentrations in the presence of 50 vol% and 75 vol% aqueous ethanol. At 50 vol% ethanol, no 4-NiP conversion is observed in the presence of 15 mM of NaBH_4_, while at 20 mM, the absorbance at 400 nm declines with time initially, although the slope of the kinetic curve gradually decreases until the reaction terminates when ∼50% 4-NiP is consumed. Full 4-NiP conversion is obtained with 25 mM of NaBH_4_, and the reaction rate is only slightly dependent on the NaBH_4_ concentration in the range of 25 to 75 mM. Note that with an increase of the ethanol content in the initial reaction mixture, the minimum amount of NaBH_4_ required for the 4-NiP reduction to proceed also increases. In the presence of 75 vol% aqueous ethanol and 25 mM of NaBH_4_ no change in the absorbance at 400 nm is detected, and full 4-NiP conversion is observed only when the borohydride concentration reaches 50 mM (Fig. 7b). This result suggests that the ratio between NaBH_4_ and alcohol in the reaction mixture is a key parameter determining the catalytic performance in aqueous alcohol mixtures.

### Effect of temperature on 4-NiP reduction kinetics in aqueous alcohol mixtures

**Fig. 8:**
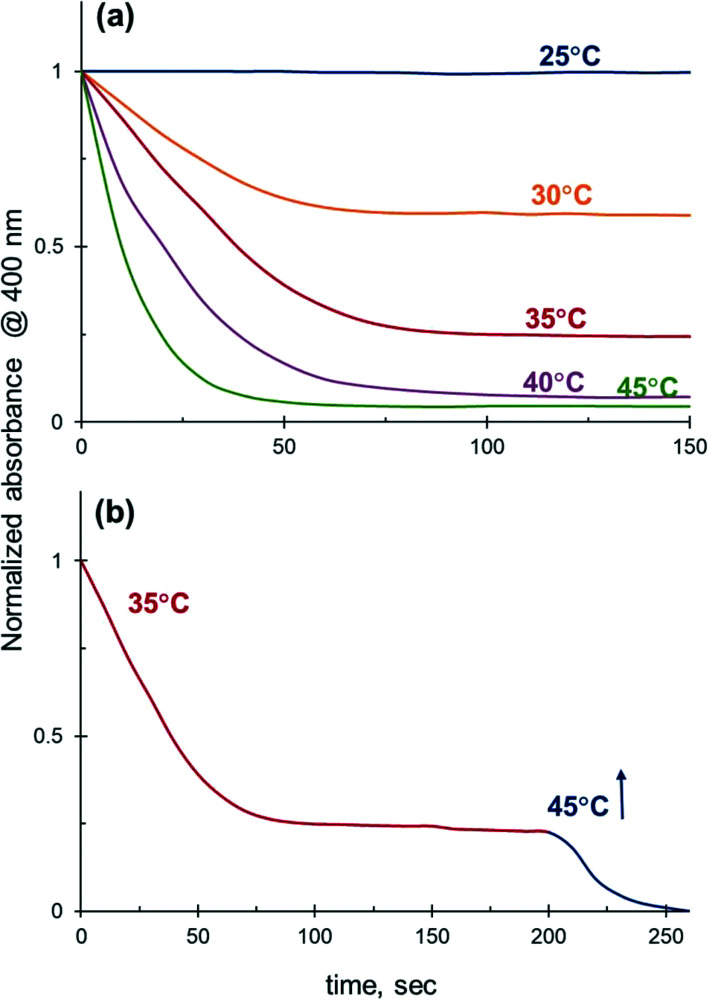
Effect of temperature on the 4-NiP reduction rate in 50 vol% aqueous ethanol. (a) The reaction performed at different initial temperatures color-coded blue, orange, red, purple and green for 25, 30, 35, 40, and 45 °C, respectively. (b) Effect of an increase in temperature from 35 to 45 °C during the course of the reaction.

The catalytic performance of Ag NPs in the reduction of 4-NiP in water–ethanol medium improves substantially with increasing reaction temperature. The 4-NiP conversion in the presence of 50 vol% aqueous ethanol rises from 0 to 100% with a temperature increase from 25 to 45 °C (Fig. 8a). The maximum attainable conversion of 4-NiP in water–ethanol medium also increases with temperature. More importantly, the observed halt of the reduction process at a certain level of conversion is not terminal. For instance, while no detectable change in 4-Nip absorption is observed after its conversion reaches ∼75% at 35 °C, further increase of the temperature to 45 °C allows for complete conversion (Fig. 8b).

**Fig. 9:**
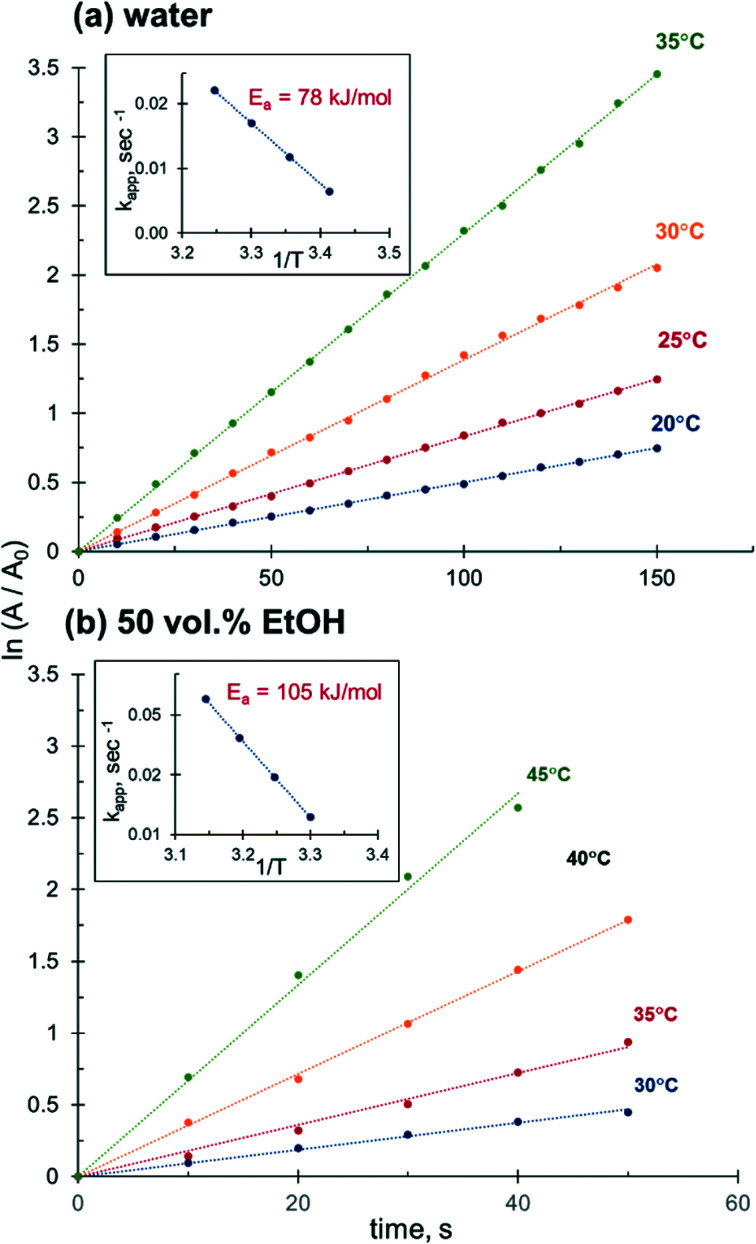
Effect of temperature on the 4-NiP reduction rate in (a) water and (b) 50 vol% aqueous ethanol.

This temperature-dependent study reveals that the apparent activation energy of 4-NiP reduction is affected by the presence of alcohol in the reaction medium. Fig. 9 shows the effect of temperature on the apparent rate constant of 4-NiP reduction in water and in 50 vol% aqueous ethanol. Arrhenius plots (Fig. 9, insets) indicate that the apparent activation energy increases from 78 kJ mol^−1^ in water to 105 kJ mol^−1^ in 50 vol% aqueous ethanol.

### Effect of pH on 4-NiP reduction kinetics in aqueous ethanol

**Fig. 10:**
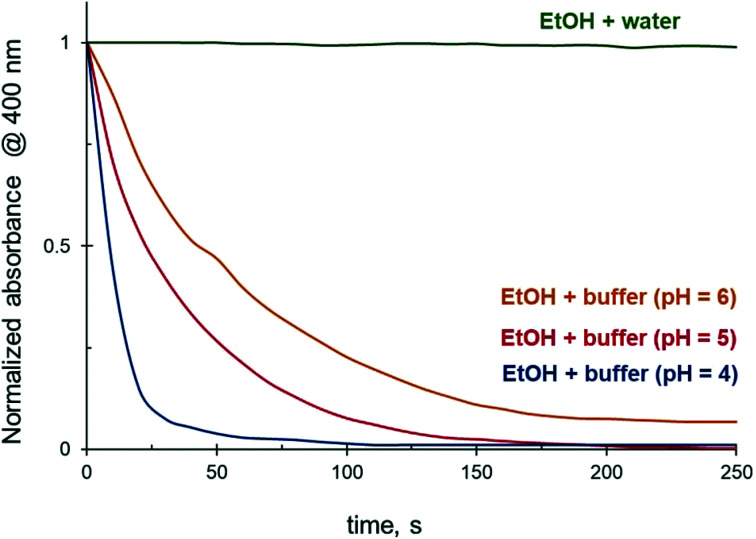
Effect of pH on the 4-NiP reduction rate in 50 vol% aqueous ethanol.

The reduction rate of 4-NiP in water–ethanol mixtures is strongly affected by the pH of the reaction medium. Using a citrate buffered 4-NiP solution in 50 vol% aqueous ethanol (Fig. 10), we demonstrate that while unbuffered solutions effectively do not react, a decrease in pH allows the reaction to proceed. The lower the pH of the buffer, the faster the full 4-NiP conversion is achieved (Fig. 10). Although a strong pH dependence of the 4-NiP reduction rate by NaBH_4_ in aqueous medium has previously been reported,^36–38^ the reason why the process is pH-sensitive is unclear. The influence of pH on the kinetics of 4-NiP reduction in both water and aqueous ethanol is addressed in more detail in the discussion.

### Effect of dissolved oxygen on 4-NiP reduction kinetics

**Fig. 11:**
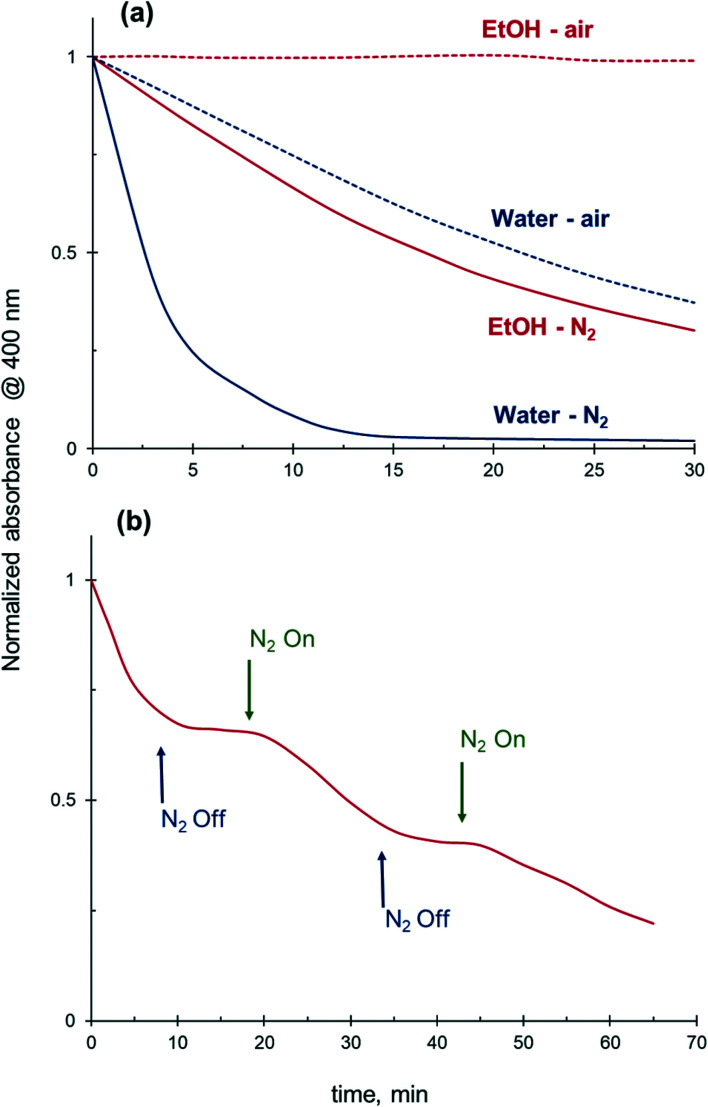
Effect of the dissolved oxygen on the 4-NiP reduction rate. (a) Reaction in water (blue) and ethanol (red) over Ag NPs. Solid line: preliminary O_2_ removal, reaction under N_2_; dashed line: no O_2_ removal, reaction under air. (b) Reaction in ethanol with and without N_2_ purging.

Removal of the dissolved oxygen from the reaction medium prior to reaction results in a substantially improved catalytic performance in both water and ethanol. Without oxygen removal, the 4-NiP absorbance only gradually decreases with time in aqueous medium and no change in the absorbance is detected in ethanol (Fig. 11a). However, when the reaction mixture is purged with N_2_ prior to the addition of NaBH_4_ and Ag NPs, a rapid decay in absorbance at 400 nm is observed in water after the catalyst addition, while in ethanol the reduction proceeds at a moderate rate. Fig. S3† shows the effect of oxygen removal on the kinetics of 4-NiP reduction in water and ethanol. The apparent rate constant increases 6 times after oxygen removal from aqueous reaction mixture (Table 3). Worth mentioning, the 4-NiP reduction rate in ethanol after oxygen removal is comparable to that obtained in water without N_2_ purging (Fig. 11a and S3†). The apparent rate constants in water under oxygen-saturated conditions and in ethanol under N_2_ atmosphere were 0.68 × 10^−3^ s^−1^ and 0.54 × 10^−3^ s^−1^, respectively (Table 3).

**Table 3:** Apparent rate constants (*k*_app_) for the 4-NiP reduction in water and ethanol in oxygen- and nitrogen saturated conditions

Catalyst	*k*_app_ × 10^3^, s^−1^			
	Water–N_2_	Water–Air	EtOH–N_2_	EtOH–air
Ag NPs	4.1	0.68	0.54	—

Due to fast oxygen uptake from the air, not only the initial oxygen content but also the reaction atmosphere is an important parameter affecting the overall catalytic performance. Indeed, while the 4-NiP reduction in ethanol proceeds with a moderate rate under N_2_ atmosphere, a rapid kinetic response was observed when the N_2_ flow was paused (Fig. 11b). Under the now oxygen-containing atmosphere, the reduction rate halts to close to zero. The observed inhibiting effect of air was partially reversible – the reduction resumed with a slightly lower rate after N_2_ had been purged through the reaction mixture for several minutes.

## Discussion

Our experimental results can be summarized as follows: (1) the addition of methanol, ethanol, or isopropanol to the reaction mixture leads to a substantial decrease in the 4-NiP reduction rate; (2) the reduction rate of 4-NiP in aqueous alcohol mixtures can be improved by increasing the borohydride concentration or the reaction temperature; and (3) the removal of dissolved oxygen from the reaction medium prior to and during the reaction results in a substantially improved catalytic performance both in water and in ethanol. The observed negative effect of methanol, ethanol, and isopropanol on the 4-NiP reduction rate is somewhat counter-intuitive since alcohols are common solvents for borohydride-mediated reductions.^39,40^ It is generally accepted that 4-Nip reduction by NaBH_4_ is not accompanied by side products formation, which is also confirmed by the presence of isosbestic points in the time-dependent absorption spectra of 4-NiP reduction (Fig. S1†). Therefore, the absence of side reactions makes it unlikely that the observed hindering effect is caused by an alteration of the reaction direction in the presence of alcohols. However, solvents are known to interact with reagents and/or reaction intermediates, substantially affecting their transformation rates.^1–11^ For example, solvent molecules can influence electron transfer kinetics or stabilize high-energy transition states, thereby accelerating the reaction. In contrast, in some cases, highly solvated reagents/intermediates are less reactive since a favourable interaction with the solvent reduces their adsorption on the catalytic active sites. Similarly, the adsorption of solvent molecules on the catalyst surface decreases the active site's accessibility for the reaction substrates. Such solvation effects are fundamentally problematic to directly address owing to the intrinsic instability of the transition states and the large number of species/interactions involved in a catalytic cycle. Hence, in the present study, we attempted to gain understanding by correlating the effect of the reaction medium on the rate of 4-NiP reduction with selected solvent properties.

### Oxygen solubility

The high oxygen affinity of metallic nanoclusters renders their structure extremely sensitive to the presence of dissolved oxygen (DO) in the surrounding medium.^44–46^ However, the effect of DO on the catalytic performance of metallic NPs is generally overlooked, with few exceptions. In a recent series of publications by Neterina and co-authors, the role of DO in the 4-NiP reduction by NaBH_4_ catalyzed by different metallic NPs was investigated.^47–49^ They showed that DO inhibits the reduction of 4-NiP, and that the reaction can only proceed once the oxygen concentration falls below a catalyst-dependent critical value. According to these authors,^47^ the inhibiting effect of DO explains the induction period commonly observed during 4-NiP reduction.^16,17,50^ The inhibiting effect of DO on the 4-NiP reduction rate was attributed to the rapid oxidation of 4-AP back into 4-NiP which stops when the DO has been consumed.^47^ More recently Maschmeyer *et al.* confirmed that DO consumption by NaBH_4_ indeed corresponds to an induction period in the kinetic profile of 4-NiP reduction over Ag NPs.^21^ However, the existence of the reverse oxidation of 4-AP into 4-NiP by DO in the presence of Ag NPs proposed by Neterina was not confirmed by other studies.^21,51^ It was suggested that DO is instead consumed both by oxidizing of the reaction intermediate (4-nitrosophenol), and by re-oxidizing the catalyst surface.^21^ The participation of DO in the catalyst surface oxidation seems plausible since Ag NPs indeed have been found to be very reactive toward oxygen in the presence of sodium borohydride.^52^

Our results confirm that DO indeed plays a critical role in 4-NiP reduction kinetics both in aqueous and alcoholic media. We showed that removing the DO prior to and during the reaction results in a substantially improved catalytic performance of Ag NPs both in water and in ethanol (Fig. 11a). Moreover, the 4-NiP reduction in ethanol proceeds with a moderate rate under N_2_ atmosphere whereas exposure of the reaction mixture to air temporarily stalls the reaction (Fig. 11b). We believe the considerably higher solubility of oxygen in alcohols compared to water (Table 4) explains, at least partially, the observed negative effect of alcohols on 4-NiP reduction kinetics. In fact, the current concentration of DO, *i.e.* at a given time, during 4-Nip reduction is defined by three main factors: (1) the initial DO concentration, (2) the rate of DO consumption, and (3) the rate of oxygen uptake from the atmosphere. If the rates of DO consumption and uptake are not affected noticeably by the presence of alcohols in the reaction mixture, an increase in the initial DO concentration due to higher oxygen solubility will inevitably lead to a higher current DO concentration and consequently lower catalytic performance. This negative effect can be compounded by an increased rate of oxygen uptake when the reaction is performed under vigorous stirring in air. A higher rate of DO consumption, on the contrary, allows to decrease the current DO concentration and, accordingly, improves catalytic performance. Our data indicate that the 4-NiP reduction rate in aqueous ethanol can be improved noticeably by increasing the NaBH_4_ concentration and/or reaction temperature (Fig. 7 and 8). In fact, in addition to a known decrease in oxygen solubility with temperature,^53^ the DO consumption rate increases with temperature and NaBH_4_ concentration.^49,51^ Furthermore, the dependence of the 4-NiP reduction rate on the ratio between NaBH_4_ and alcohol observed in this study (Fig. 7) is also consistent with the hypothesis that alcohols are responsible for the increase of the current concentration of DO in the reaction mixture. To allow the reaction to proceed with the same rate, the negative effect of an increased initial DO levels due to a higher alcohol content should be counterbalanced by a higher rate of DO consumption at increased NaBH_4_ concentrations. Finally, the results of the current study, showing that the inhibiting effect of alcohols was more pronounced for Ag than for Au NPs, is also in good agreement with a higher reactivity of Ag NPs towards oxidation by DO, which makes reduction in the presence of Ag NPs more sensitive to its concentration.

**Table 4:** Solvent properties: viscosity at 298.15 K (*μ*), polarity (*E*_T_ (30)), ratio polarity/polarizability (π*), acidity (p*K*_a_), and solubility of oxygen at 298.15 K and 101.3 kPa (*X*_G_)

Solvent	*μ*,^41^ mPa s	*E*_T_ (30)^42^	π* (ref. 42)	p*K*_a_ (ref. 41)	*X*_G_ (O_2_),^43^ molar fraction
H_2_O	0.89	63.1	109	15.7	4.70^a^
MeOH	0.54	55.4	60	15.5	41.5
EtOH	1.07	51.9	54	16.0	57.1
IPA	2.04	49.2	52	17.1	77.8

a Data from ref. 41.

It could be argued that the addition of only 25 vol% of alcohol should not significantly change the amount of DO in the reaction medium and accordingly impinge on the 4-NiP reduction kinetics to the extent observed in our study (Fig. 4). Unfortunately, the data on oxygen solubility in aqueous alcohol mixtures are very sparse and controversial.^53–55^ However, according to ref. 21 the addition of even a relatively small amount of oxygen by injection of an oxygen-saturated aliquot of water during aqueous 4-NiP reduction caused a severe disruption to the reduction process. Also, the oxygen solubility in aqueous alcohol mixtures is not simply an interpolation of its solubilities in pure liquids since the addition of alcohol to water substantially affects the solvent structure. Yet, the kinetic trends observed here cannot be explained only by the difference in oxygen solubilities in water and alcohols. In particular, the rate of 4-NiP reduction in ethanol was slower compared to water even after removal of DO by purging N_2_ through the reaction mixture (Fig. 11). More importantly, the difference in oxygen solubilities cannot explain the increase in apparent activation energy of 4-NiP reduction in the aqueous ethanol mixtures which should be independent of the DO concentration. Therefore, additional factors must be considered.

### Solvent polarity

Solvent polarity is known to have a significant effect on the rates of catalytic reactions.^1,2^ According to Hughes–Ingold rules – a simple solvation model which takes in account only electrostatic interactions between initial and transition states with solvents – an increase in solvent polarity accelerates rates of reactions where a charge develops in the activated complex from a neutral or slightly charged reactant. On the contrary, the rates of reactions where the charge density of the activated complex is lower than that of the initial substrate decreases with solvent polarity. The effect of the solvent polarity on the rate of 4-NiP reduction with H_2_ over Pt/C catalyst^56^ and photocatalytic reduction in TiO_2_ suspension^57^ was previously reported. Vaidya *et al.*^56^ investigated the former in various solvents including water, methanol, ethanol, *n*-propanol, and *n*-butanol. It was demonstrated that the average catalytic activity and the initial rate of hydrogenation increased with an increase in the solvent polarity and were highest in water. The enhancement in the reaction rate was attributed to the increase in the activity of nitro compounds in polar solvents. Brezova *et al.*^57^ demonstrated that the rate of photosensitized reduction of 4-NiP also increased with increasing solvent polarity. According to the authors, the rate of back electron transfer from nitro radical anions to the TiO_2_ valence band efficiency decreases with the increase in solvent polarity. Since 4-NiP reduction with NaBH_4_ is a complex process that includes sequential electron transfer steps,^58^ the solvent polarity is expected to have a similar effect on the reaction kinetics; namely, the 4-NiP reduction is expected to proceed more efficiently in a more polar solvent. The difference in the polarities of water and alcohols (Table 4) could thus in part explain the lower rate of 4-NiP reduction in aqueous alcohol mixtures, where the alcohol addition decreases the polarity.

The ability of the solvent to stabilize the charged intermediates can be also described by the parameter π* – a ratio between polarity and polarizability.^59–61^ As can be seen from Table 4, this parameter indeed correlates well with the reaction performance in the studied solvents, and therefore can be considered as one of the factors responsible for the observed inhibiting effect of alcohols on the reaction kinetics.

### Viscosity

The viscosity of the reaction medium can significantly influence the rate of heterogeneous catalytic reaction. Particularly, viscosity directly affects the diffusion of reactants to and from the catalytic active sites. According to the Stokes–Einstein equation, the diffusion coefficient (*D*) in a liquid medium is inversely proportional to the dynamic viscosity (*η*):

1 *D* = *k*_B_*T*/6π*rη*

where *k*_B_ is Boltzmann's constant, *T* is the absolute temperature, and *r* is the radius of the diffusing species approximated by a sphere.

Although it is generally assumed that aqueous 4-NiP reduction proceeds under a kinetically controlled regime, where the diffusion of the reactants to and from the NPs surface is fast,^19,62^ in some cases the diffusion and adsorption rates of the reactants can be considered important factors affecting 4-NiP reduction rate.^50,63–65^ A change in the viscosity of the reaction medium can create additional diffusional barriers and therefore decrease the overall reaction rate. Experimental results obtained in this work demonstrate that under conditions corresponding to an efficient 4-NiP reduction in an aqueous medium, the reaction is hindered in the presence of >50% of alcohol (Fig. 4). The data on dynamic viscosities of water and alcohols (Table 4) indicate that the difference in viscosities of the pure solvents is unlikely responsible for the observed inhibiting effect. Indeed, the viscosity of ethanol is only slightly higher than that of water, while methanol has the lowest viscosity among the studied solvents. However, the physical properties of binary mixtures often deviate from ideal mixing, and a positive deviation of viscosity is observed in the case of water–alcohol mixtures.^66–68^ The viscosity of binary mixtures of water with methanol, ethanol, or isopropanol increases noticeably with an increasing mole fraction of water. The maximum value, substantially larger than either of the constituents, is reached at around 70 vol% water.^68^ More importantly, the maximum viscosity values increase from 1.73 mPa s for water–methanol to 2.38, and 2.85 for water–ethanol, and water–isopropanol mixtures, respectively. Our results showing an increase in effect with increased molecular weight of the alcohol are in line with the viscosity increase trend. Therefore, viscosity cannot be ruled out as a contributor to the negative effect of alcohols on the 4-NiP reduction rate, at least for the case when the reaction is performed in binary water–alcohol mixtures.

### Chemical interaction

The involvement of the solvent in the chemical transformations accompanying catalytic processes is another plausible explanation for the solvent effects on the reaction rate. Indeed, solvent molecules can be involved in a catalytic cycle, *e.g.* serve as a substrate or participate in activation of reactants. This interaction between solvent and reaction substrates or catalyst active sites can lead to the formation of active species capable of further activation of reactants, or, alternatively, provide new reaction pathways. Choi *et al.*,^69^ demonstrated that 4-NiP reduction by NaBH_4_ in the presence of Ag NPs proceeded more efficiently when the aqueous borohydride solution was aged. They assumed that the negative charge of borohydride prevented it from approaching the negatively charged NPs, resulting in a slower reaction rate. Meanwhile, hydrolysis of borohydride leads to the formation of neutrally charged borane intermediates believed to react with NPs instantly. Although partially hydrolysed borohydride species, which mass matched the neutral intermediates, were detected by mass spectrometry, there is no direct evidence that those intermediates were indeed involved in the 4-NiP reduction catalytic cycle. The importance of this NaBH_4_ hydrolysis step during 4-NiP reduction was reported in another, recent, paper,^36^ which states that 4-NiP reduction by NaBH_4_ in an aqueous medium in presence of metallic NPs proceeds over two competing pathways: slower borohydride-induced and faster hydrogen-induced. The authors speculated that hydrolysis of NaBH_4_ in aqueous media accompanied by hydrogen gas formation substantially improves the rate of 4-NiP reduction. Based on the fact that NaBH_4_ hydrolysis is a pH-dependent process,^70^ they concluded that an increase in the 4-NiP reduction rate with a decrease of pH can be attributed to a higher rate of NaBH_4_ hydrolysis.^36^ Note that hydrogen generation from NaBH_4_ can be performed both by hydrolysis and alcoholysis. The highest hydrogen generation rate is observed during methanolysis^71,72^ due to its higher acidity compared to water and other alcohols (Table 4). In particular, at 25 °C the methanolysis rate is 10 times faster than hydrolysis.^71^ Assuming a faster hydrogen-mediated pathway during 4-NiP reduction, it can be expected that the reaction would proceed more efficiently in methanol than in water. However, our data indicate that 4-NiP reduction is suppressed in methanol. This implies that the solvent interaction with NaBH_4_ accompanied by H_2_ formation (hydrogen-mediated pathway) plays a negligible role during the catalytic reduction of 4-NiP with NaBH_4_, at least when the reaction is performed in the presence of alcohols. At the same time, our data indicate that the 4-NiP reduction rate in water–ethanol mixtures is also strongly affected by the pH of the reaction medium (Fig. 10). Therefore, an alternative explanation of the role of the pH of the reaction medium in 4-NiP reduction is needed.

According to Fountoulaki *et al.*,^73^ the ability of water to donate protons plays a crucial role in the reduction rate of nitroarenes by NaBH_4_. We believe that the accessibility of protons during the 4-NiP reduction is indeed an important factor that affects the rate and is influenced by the pH of the reaction medium. In particular, we know that the concentration of DO in the reacting mixture strongly affects the overall 4-NiP reduction rate (see above). Meanwhile, DO concentration is affected by its consumption by the borohydride, *i.e.* BH_4_^−^ + 2O_2_ + H^+^ → H_3_BO_3_ + H_2_O, the effectiveness of which is strongly pH-dependent. Accordingly, an increase in DO depletion with a decrease in pH leads to the improvement of the overall 4-NiP reduction rate.

In addition to an apparent effect of the pH on the DO reduction rate by NaBH_4_, the possibility of direct involvement of H^+^ in the 4-NiP reduction catalytic cycle should also be considered as a contributor to the observed higher 4-NiP reduction rate at lower pH. Recently, Kong *et al.*,^74^ studied the mechanism of 4-NiP reduction by NaBH_4_ based on the recognized reaction intermediates using paper assisted ultrasonic spray ionization mass spectrometry (PAUSI-MS). Isotope analysis showed the hydrogen atoms of the amine group of the 4-AP originated from water molecules and not from BH_4_^−^. Based on these findings, the authors suggested that water molecules serve as the only hydrogen source during 4-NiP reduction while NaBH_4_ contributes to the production of H^+^ from water molecules. While there remain questions about the role of the catalyst, this study firmly indicates that the role of water in 4-NiP reduction catalytic cycle is beyond just that of a reaction medium.

## Conclusions

The correlation between the properties of the reaction medium and catalytic performance is an important factor often overlooked in the literature. Here, the effect of solvent on the kinetics of the liquid phase 4-NiP reduction by NaBH_4_ over Au and Ag NPs has been investigated. We demonstrated that the presence of methanol, ethanol, or isopropanol in the reaction mixture drastically affects the reduction kinetics. We showed that, at typical conditions, the 4-NiP reduction is completely suppressed by the presence of 50 vol% of aqueous ethanol. The observed hindering effect exhibits a strong temperature and NaBH_4_ concentration dependence. In particular, the reduction rate in the presence of alcohols improves noticeably with an increase in temperature and sodium borohydride concentration. Our analysis demonstrates that the difference in oxygen solubilities in water and alcohols is among the factors responsible for the observed effects. However, some of the kinetic features, reported in this study, such as the increase in the apparent activation energy of 4-NiP reduction in aqueous ethanol mixtures cannot be explained only by an increase in dissolved oxygen concentration upon alcohol addition. While the correlations between solvent properties and the reaction performance discussed here are essentially qualitative, our results have at least two important implications. First, our experimental data confirm that solvent properties, including oxygen solubility, strongly affect the catalytic performance of metallic NPs in the liquid phase 4-NiP reduction. Therefore, standardization of the reaction conditions which include preliminary removal of the dissolved oxygen is essential for an unambiguous comparison of the catalytic performance of metallic NPs. Second, since the 4-NiP reduction with NaBH_4_ is considered an intermediate step in industrial wastewater treatment and commercially relevant synthetic pathway, the effects of solvent on the catalytic performance reported in this work must be considered to design the overall process. Although increasing the reaction temperature, sodium borohydride concentration, and preliminary removal of dissolved oxygen substantially improve the catalytic performance both in water and water–alcohol mixtures, such approaches incurs additional costs. Furthermore, the final borohydride concentration in the treated wastewater must also be considered. Thus, the composition of the reaction medium needs to be taken into account in order to obtain comparable conditions and maximize process efficiency.

## Author contributions

All authors were involved in planning the experiments. V. L. performed catalytic experiments, V. L. and J. A. synthesized nanoparticles, J. A. and E. R. performed electron microscopy. All authors contributed to writing and editing the manuscript.

## Conflicts of interest

The authors report no competing interests.

## Supplementary Material

RE-007-D2RE00044J-s001
