# Supplementary material for: Solvent effects on the kinetics of 4-nitrophenol reduction by NaBH4 in the presence of Ag and Au nanoparticles
Source: React Chem Eng. 2022 Apr 29;7(8):1728–41. doi: 10.1039/d2re00044j (PMC9316932; doi:10.1039/d2re00044j)
Supplement: RE-007-D2RE00044J-s001 [file RE-007-D2RE00044J-s001.pdf]

Electronic Supplementary Material (ESI) for Reaction Chemistry & Engineering.
This journal is © The Royal Society of Chemistry 2022

Supplementary Information

Solvent Effects on the Kinetics of 4-Nitrophenol Reduction by $NaBH_4$ in the Presence of Ag and Au Nanoparticles

Vladimir Lomonosov,[1,2] Jérémie Asselin,[1,2] Emilie Ringe[1,2]*

1. Department of Materials Science and Metallurgy, University of Cambridge, 27 Charles Babbage Road, Cambridge, United Kingdom, CB3 0FS
2. Department of Earth Sciences, University of Cambridge, Downing Street, Cambridge, United Kingdom, CB2 3EQ

* Corresponding author: er407@cam.ac.uk; +44 (0)1223 334330 (ph.), +44 (0)1223 334567 (fax).

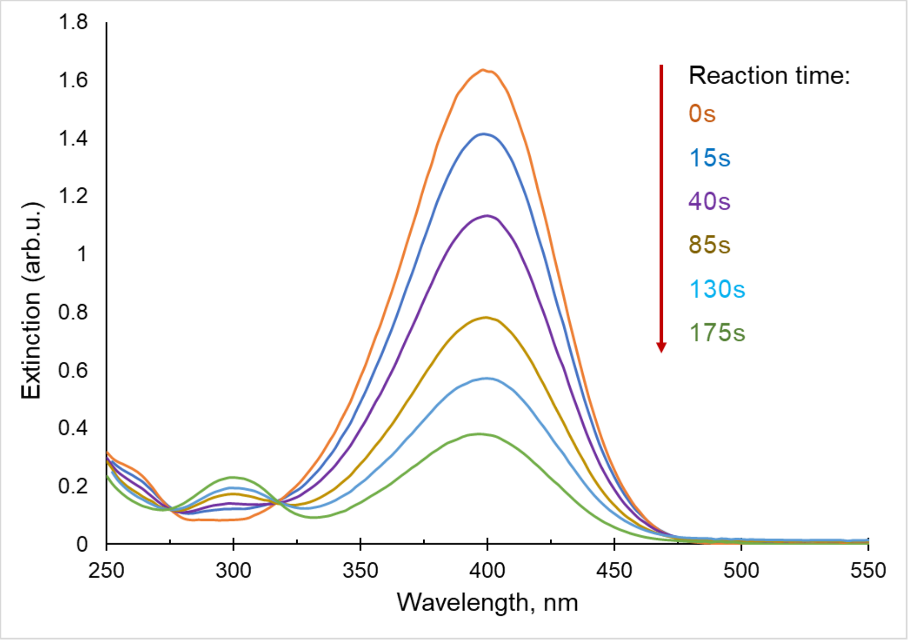

Figure S1. Time-dependent absorption spectra of the 4-NiP (0.1mM) reduction by $NaBH_4$ (15 mM) in aqueous medium in the presence of Ag NPs (1.5×10^{-3} mM).

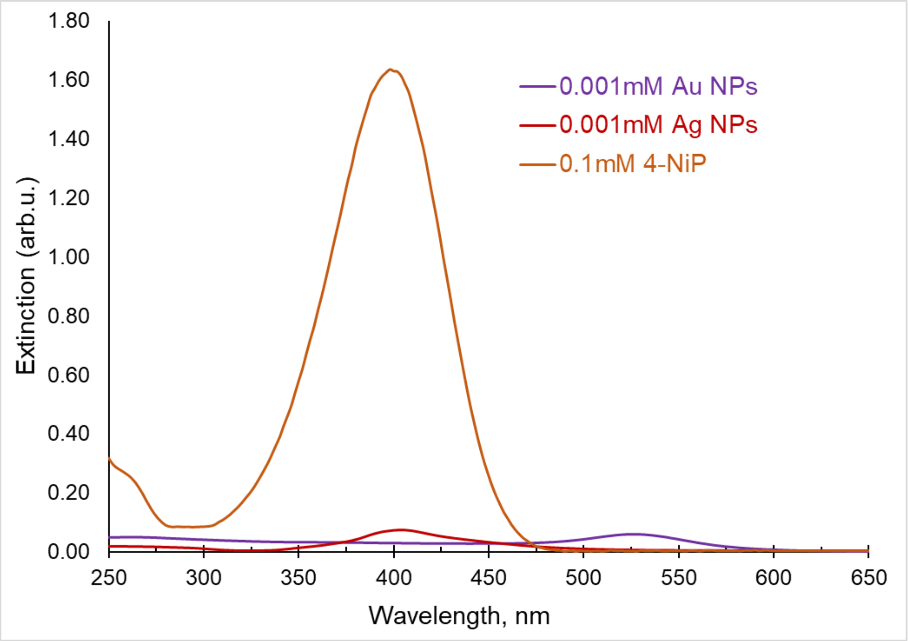

Figure S2. Extiction spectrum of 0.1mM of 4-nitrophenolate anion and 0.001 mM of Au and Ag NPs.

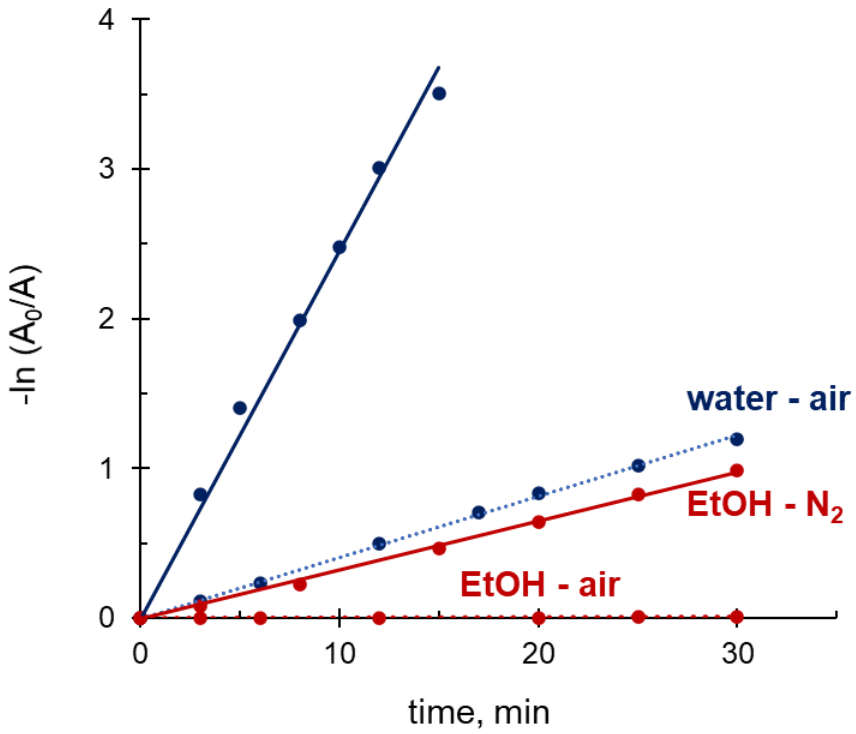

Figure S3. Effect of the dissolved oxygen on the 4-NiP reduction kinetics. Reaction in water (blue) and ethanol (red) over Ag NPs. Solid line: preliminary O_2 removal, reaction under N2; dashed line: no O2 removal, reaction under air.
